# A Prospective Comparative Study of Postoperative Pain and Functional Outcomes With and Without Cryotherapy Following Total Knee Arthroplasty

**DOI:** 10.7759/cureus.85227

**Published:** 2025-06-02

**Authors:** Ali Tauseef, Muhammad Mannan, Nayan Shrivastava, Muhammad A Hamid, Raza Mehmood, Fayyaz Ur Rahman Haider, Khalil Farooque

**Affiliations:** 1 Trauma and Orthopaedics, University Hospital Birmingham NHS Foundation Trust, Birmingham, GBR; 2 Trauma and Orthopaedics, Liaquat National Hospital and Medical College, Karachi, PAK; 3 Orthopaedic Surgery, University Hospital Birmingham NHS Foundation Trust, Birmingham, GBR; 4 Orthopaedic Surgery, Shaukat Khanum Memorial Cancer Hospital and Research Centre, Lahore, PAK

**Keywords:** cryotherapy, functional outcome, knee society score (kss), postoperative pain, total knee arthroplasty (tka), visual analog scale (vas)

## Abstract

Background

Total knee arthroplasty (TKA) is a major orthopedic procedure often associated with significant postoperative pain, inflammation, and reduced range of motion (ROM). Effective pain management and functional recovery remain key challenges in postoperative care. This study aims to evaluate the impact of cryotherapy on postoperative pain and range of motion (primary outcomes) and to assess functional improvement using the Knee Society Score (KSS; secondary outcome) in patients undergoing total knee arthroplasty. This prospective study was conducted in the Department of Orthopaedic Surgery, Liaquat National Hospital, Karachi, from November 26, 2020, to May 25, 2021.

Materials and methods

A total of 194 patients were included in the study using consecutive non-probability sampling. Patients were divided into Group A (with cryotherapy) and Group B (without cryotherapy). Clinical evaluations were performed at baseline, on the fifth postoperative day, the second week, the sixth week, and the twelfth week. Primary outcomes included the visual analogue scale (VAS) for pain and range of motion (ROM), while the secondary outcome was the KSS. Stratification and chi-square tests (p < 0.05 as significant) were applied.

Results

In the twelfth week, the VAS score in Group A was 0.00 ± 0.00, indicating complete resolution of pain in all patients. In contrast, Group B reported a slightly higher mean score of 0.06 ± 0.24. Although this difference was clinically relevant, it was not statistically significant (p = 0.067). ROM was 117.92 ± 13.85 in Group A versus 114.57 ± 13.54 in Group B (p = 0.041). Knee scores were 74.46 ± 11.32 in Group A and 72.54 ± 10.43 in Group B (p = 0.029), while functional scores were 76.38 ± 10.37 in Group A and 75.18 ± 10.40 in Group B (p = 0.029). Significant differences in VAS and ROM were observed at specific time points between Group A and Group B.

Conclusion

The study concluded that cryotherapy significantly improved pain scores, ROM, and knee scores in patients with TKA compared to those without cryotherapy.

## Introduction

Total knee arthroplasty (TKA) is a major orthopaedic procedure often associated with tissue damage, inflammatory responses, and pain [1-3]. There is also significant blood loss, which can be upto 1.5 L, requiring transfusions in 10-15% of patients. Secondary events include local swelling and oedema, reduced range of motion (ROM), stiffness, and reduced quadriceps strength. Despite advances in anaesthetic techniques and multimodal pain management, TKA remains challenging for most patients [1,4,5].

Cryotherapy has been described since the time of Hippocrates [6]. It is widely used after sports trauma as an emergency treatment and postoperatively after anterior cruciate ligament (ACL) reconstruction. Local application of cold penetrates up to 4 cm below the skin. This causes vasoconstriction and alters tissue metabolism by decreasing enzymatic function [7,8]. The reduced blood flow decreases the local inflammatory response and oedema formation [8]. The anaesthetic effect of local cooling is produced by slowing or eliminating the transmission of pain signals [9]. Furthermore, cold inhibits the stretch reflex and reduces muscle spasms [8]. Despite these physiological effects being well-established, consistent clinical benefits following major joint surgeries such as TKA remain uncertain. Cold application alone has a limited effect on swelling and may actually increase it due to cold-induced ischaemic damage to blood vessels [10]. Simultaneous application of cold and compression is more effective, as compression synergistically reduces blood flow and oedema formation. It also provides support to soft tissues [11,12].

Several cryotherapy options are available, including first-generation cold therapy, such as crushed ice in a plastic bag or cold/gel packs; second-generation therapy using circulating ice water with or without compression, such as Cryo/Cuff (Aircast, Vista, CA, USA) or Game Ready (Cool Systems Inc., Concord, CA, USA); and third-generation advanced computer-assisted devices with continuous controlled cold therapy (e.g., cTreatment, Waegener, Beerse, Belgium, and CTM 5000, Ener-C AG, Baar, Switzerland). The advantage of these devices is controlled temperature modulation with continuous cooling at a specific temperature (11 °C) for prolonged durations. They prevent cold-induced vasodilation and are less labour-intensive, as they do not require frequent refilling with cold water or ice [13].

Cryotherapy is the standard of care in some countries but is rarely used in others [14]. This is the result of conflicting evidence from different randomised trials [1]. While some studies show benefits, others demonstrate little to no improvement, particularly in outcomes relevant to TKA. Much of the earlier evidence is from arthroscopic or soft tissue procedures, which differ significantly from TKA in terms of invasiveness and recovery trajectory. Third-generation cryotherapy providers attribute this lack of evidence to inappropriate cooling techniques. They advocate for value-added devices at a significant financial cost. Studies on cryotherapy after TKA have been limited and controversial. Earlier research on the use of cryotherapy after arthroscopic knee surgery showed mixed results [15,16].

Cohn et al. studied 26 patients who underwent ACL reconstruction and received continuous cryotherapy via a thermal blanket for 4 days postoperatively [15]. Patients in the cryotherapy group required significantly less analgesic medication during hospitalisation, but pain outcome measures like the visual analogue scale (VAS) or McGill Pain Questionnaire were not included [17]. Daniel et al. examined 151 patients with post-arthroscopic ACL reconstruction with or without meniscal repair. While cooling pads were incorporated into dressings, no significant differences were observed between groups for knee girth, ROM, pain, or medication use during the three-day hospital stay [16].

The benefits of cryotherapy after total knee joint replacement surgery have also been reported. Hecht et al. demonstrated decreased knee swelling following 10 cryotherapy sessions, while Williams et al. found less use of analgesics in patients receiving cryotherapy with continuous passive motion versus continuous passive motion alone [15,18-20]. However, neither study used standardised pain outcome variables. Different methods of cryotherapy application have been explored such as ice chips or gel packs. Ice chips applied directly were more effective in reducing skin temperature than gel packs [21]. Oosterveld et al. demonstrated a significant decrease in intra-articular knee temperature in normal subjects following a 30-minute ice chip application. Even after a routine recommendation of three to four cryotherapy sessions per day, significant clinical improvements in pain and symptoms are rarely reported postoperatively [22]. Given these inconsistencies, further evidence is required to assess whether cryotherapy can meaningfully improve recovery in TKA patients, particularly in terms of pain reduction, range of motion, and functional recovery.

This study aims to evaluate the impact of cryotherapy on postoperative pain and range of motion (ROM) as primary outcomes, assessed using the VAS and goniometric measurements, respectively, in patients undergoing TKA. Additionally, it seeks to assess functional improvement as a secondary outcome using the Knee Society Score (KSS) to provide comprehensive evidence for cryotherapy’s role in enhancing postoperative recovery following TKA.

## Materials and methods

This six-month, prospective, non-randomised controlled cohort study was conducted at the Department of Orthopaedics, Liaquat National Hospital, Karachi, from 26-11-2020 to 25-5-2021. The sample size was 194 (n = 97 in each group) as estimated by using the WHO sample size calculator for a confidence level of 95% and a power of 80%, with an established rate of improved ROM to 90 degrees in the treatment group (80%) and the control group (64%). Consecutive non-probability sampling was employed since the constraint of time did not allow for the use of randomisation or blinding. We accept that this represents a degree of selection and performance bias, which is a limitation of our study design.

Both men and women between 50 and 80 years, who had primary unilateral or bilateral TKA, were included. Exclusion criteria included revision TKA, refusal to participate/provide informed consent, bleeding disorders (e.g., haemophilia, platelet disorders), significant intraoperative complications (e.g., fracture or neurovascular injury), and neuromuscular disorders (e.g., polio, cerebral palsy, muscular dystrophy, or Charcot's joint).

Patients who presented to the orthopaedic outpatient department and fulfilled the inclusion criteria were recruited after approval from the College of Physicians and Surgeons, Pakistan. Written Informed consent was taken from all the patients. Patients were divided into two groups: Group A underwent cryotherapy postoperatively, and Group B did not. There was no placebo given to the control group, which might have resulted in a placebo effect for Group A.

Standard primary TKA implants were used in both groups, which were operated on by experienced arthroplasty surgeons through a midline parapatellar approach. All the patients received combined spinal epidural (CSE) anaesthesia. Post-operative analgesia and physical therapy protocols were uniform among both groups and composed of one-on-one physiotherapy twice daily and walking as assisted by a mobility technician.

Patients of group A received cryotherapy sessions of 20 minutes each. It was done on a daily basis from the day of surgery till the fifth postoperative day and then continued at home for another two weeks. A second-generation continuous-flow cryotherapy device (Cool Systems Inc., Game Ready®) was used that delivers a cooling temperature of about 11°C through a circumferential knee wrap. The device has both cooling and intermittent compression effects. The session duration of 20 minutes set in this study is shorter than some earlier trials, but this protocol was chosen because of clinical feasibility, patient comfort, and institutional practice patterns.

Patients were checked at baseline, on the fifth postoperative day, and then on the second-week, sixth-week, and twelfth-week follow-ups. Pain was measured using the VAS, ROM was recorded using a goniometer, and function was assessed using the KSS. VAS and ROM were set as primary outcomes, while KSS was the secondary outcome.

A proforma was used to collect the patient's data, including age, gender, height, weight, BMI, comorbidities (hypertension, diabetes, ischaemic heart disease, chronic kidney disease), operative side, and treatment group. We observed baseline imbalances in height and weight between groups; to address this, we performed stratification and controlled for these variables in the post-stratification analysis. Frequencies and percentages were calculated for categorical variables, and means ± SD for continuous variables. Stratification was applied for key modifiers (e.g., age, gender, BMI). Associations between variables were tested using chi-square tests for categorical and t-tests for continuous data, with a p-value < 0.05 considered statistically significant.

## Results

In the overall demographic distribution, both groups were comparable, but certain baseline imbalances were noted. There were higher mean values for age, weight, height, and BMI in Group A as compared to Group B. Gender distribution remained balanced with a slight male predominance in both groups, but significant differences in comorbidity prevalence were observed. Diabetes mellitus was more common in Group B, whereas chronic kidney disease was more frequent in Group A. This is presented in Table 1.

**Table 1:** Demographics and descriptive statistics

Parameter	Group A (n = 97) – Mean ± SD	Group A – Median (Min–Max)	Group B (n = 97) – Mean ± SD	Group B – Median (Min–Max)
Age (years)	65.03 ± 9.65	64.11 (45–80)	61.23 ± 15.56	65.84 (26–80)
Weight (kg)	96.36 ± 11.16	98 (71–119)	79.05 ± 12.18	74 (59–109)
Height (cm)	196.43 ± 14.55	195 (168–227)	179.59 ± 15.41	178 (145–225)
BMI (kg/m²)	25.18 ± 3.58	24.98 (18.7–34.6)	24.77 ± 4.35	24.02 (18.7–37.6)
Gender – Male	55 (56.7%)	–	52 (53.6%)	–
Gender – Female	42 (43.3%)	–	45 (46.4%)	–

The distribution of comorbidities across both groups shows a comparable burden of hypertension, ischaemic heart disease, and chronic kidney disease. However, diabetes mellitus appeared more frequently in Group B, while chronic kidney disease was more prevalent in Group A. These baseline differences were carefully considered during the analysis. Assumptions for parametric testing were assessed by evaluating data normality and dispersion using means, standard deviations, and confidence intervals where applicable. This ensured that comparisons between groups, particularly for functional outcomes, remained valid despite observed imbalances. These variations in comorbidities are important to acknowledge, as they may affect recovery trajectories, pain perception, and overall postoperative outcomes following total knee arthroplasty. This is presented in Table 2.

**Table 2:** Comorbidities and surgery site

Parameter	Group A (Yes)	Group A (No)	Group B (Yes)	Group B (No)
Hypertension	41 (42.3%)	56 (57.7%)	36 (37.1%)	61 (62.9%)
Diabetes Mellitus	52 (53.6%)	45 (46.4%)	65 (67%)	32 (33%)
Ischemic Heart Disease	41 (42.3%)	56 (57.7%)	36 (37.1%)	61 (62.9%)
Chronic Kidney Disease	47 (48.5%)	50 (51.5%)	22 (22.7%)	75 (77.3%)

In assessing the surgical distribution between the two groups, both unilateral and bilateral procedures were well-represented. Group A included slightly fewer unilateral cases and more bilateral cases compared to Group B. This comparable distribution suggests that the functional benefits observed in the cryotherapy group were not biased by surgery type. Rather, the observed improvements in pain relief, range of motion, and knee function can be reasonably attributed to the therapeutic effect of cryotherapy itself. This is presented in Table 3.

**Table 3:** Surgery site

Surgery Type	Group A	Group B
Unilateral (%)	52 (53.6%)	59 (60.8%)
Bilateral (%)	45 (46.4%)	38 (39.2%)

At baseline, both groups experienced similar levels of postoperative pain, establishing a uniform starting point for comparison. Over the follow-up period, patients in the cryotherapy group demonstrated a more pronounced and consistent reduction in pain compared to those who did not receive cryotherapy. The most noticeable difference was observed early in the recovery process, particularly by the fifth postoperative day, where pain relief was more substantial in the cryotherapy group. While pain scores in both groups continued to decline over time, the cryotherapy group consistently reported lower values at most follow-up intervals. By the end of the twelfth week, pain had nearly resolved in both groups, though cryotherapy patients reached this point slightly earlier. These trends suggest that cryotherapy may accelerate early postoperative pain control, potentially enhancing patient comfort and participation in rehabilitation. This is presented in Table 4.

**Table 4:** Descriptive statistics of the visual analogue score (VAS) The independent samples t-test was used to calculate the p-values.

Time Point	Group A (Mean ± SD)	Group A Median (Min–Max)	Group B (Mean ± SD)	Group B Median (Min–Max)	t-value	P-Value
At Baseline	6.23 ± 2.15	5.00 (3–9)	6.04 ± 1.92	6.25 (3–9)	0.61	0.543
At 5th Day	3.17 ± 2.04	2.08 (1–6)	4.51 ± 1.24	4.69 (2–6)	2.30	0.023
At 2nd Week	1.53 ± 0.54	1.46 (1–2)	1.56 ± 0.50	2.00 (1–2)	0.24	0.812
At 6th Week	0.50 ± 0.59	0.00 (0–2)	0.87 ± 0.70	1.00 (0–2)	2.01	0.048
At 12th Week	0.00 ± 0.00	0.00 (0–0)	0.06 ± 0.24	0.00 (0–1)	1.84	0.067

Baseline ROM was nearly identical between the two groups, providing a consistent foundation for evaluating postoperative improvement. As recovery progressed, patients in the cryotherapy group demonstrated a more favourable trajectory of ROM enhancement at each follow-up point. Notably, early improvements were more marked in this group, suggesting that cryotherapy contributed to accelerated joint mobility during the critical initial weeks of rehabilitation. Although the mid-recovery period showed only marginal differences, cryotherapy patients regained a slightly higher degree of knee flexion by the 12th week. Overall, these findings indicate that cryotherapy may promote better functional outcomes by supporting faster gains in joint movement, particularly in the early postoperative phase. This is presented in Table 5.

**Table 5:** Descriptive statistics of range of motion (ROM) Statistical test used: independent samples t-test

Time Point	Group A (Mean ± SD)	Group A Median (Min–Max)	Group B (Mean ± SD)	Group B Median (Min–Max)	t-value	P-Value
At Baseline	54.96 ± 8.61	52.70 (39–73)	54.44 ± 9.11	53.32 (39–74)	0.42	0.672
At 5th Day	78.85 ± 14.53	81.14 (50–99)	74.14 ± 13.96	73.64 (49–99)	2.03	0.045
At 2nd Week	85.14 ± 9.99	83.38 (61–100)	81.88 ± 9.22	80.23 (63–100)	2.14	0.034
At 6th Week	104.98 ± 7.39	103.04 (93–118)	103.49 ± 6.96	100.22 (91–120)	1.95	0.054
At 12th Week	117.92 ± 13.85	122.04 (98–137)	114.57 ± 13.54	105.22 (96–140)	2.07	0.041

Both groups began the study with comparable KSS, indicating a uniform level of preoperative knee function. Throughout the follow-up period, both groups exhibited marked improvements in knee scores, reflecting effective surgical and rehabilitative outcomes. However, patients in the cryotherapy group consistently demonstrated slightly greater functional gains at each time point. Although the differences were modest, the steady advantage observed in this group suggests that cryotherapy may play a contributory role in optimising knee joint recovery. These findings reinforce the potential of cryotherapy to enhance the functional trajectory post-TKA when used alongside standard rehabilitation protocols. This is presented in Table 6.

**Table 6:** Descriptive statistics of the knee score Statistical test used: independent samples t-test

Time Point	Group A (Mean ± SD)	Group A Median (Min–Max)	Group B (Mean ± SD)	Group B Median (Min–Max)	t-value	P-Value
At Baseline	32.06 ± 9.56	31.30 (16–50)	31.35 ± 8.11	28.92 (16–51)	0.54	0.621
At 5th Day	50.21 ± 10.39	49.17 (30–68)	48.70 ± 9.04	47.04 (32–68)	2.01	0.048
At 2nd Week	68.88 ± 10.64	68.17 (49–90)	67.35 ± 9.32	65.28 (51–94)	2.14	0.036
At 6th Week	70.41 ± 10.69	69.70 (51–92)	68.91 ± 9.39	66.84 (53–96)	2.07	0.041
At 12th Week	74.46 ± 11.32	73.58 (53–98)	72.54 ± 10.43	71.21 (54–102)	2.19	0.029

Baseline functional scores were similar between the two groups, indicating comparable preoperative activity levels and mobility. Over the course of recovery, both groups demonstrated progressive functional improvement, reflecting effective surgical intervention and rehabilitation. Notably, patients in the cryotherapy group consistently reported slightly better functional scores at each follow-up interval. Although the differences were relatively modest, this consistent pattern suggests that cryotherapy may offer a supportive benefit in enhancing overall physical function during the postoperative period. Its role appears to be particularly valuable in promoting early mobility and sustaining functional recovery over time. This is presented in Table 7.

**Table 7:** Descriptive statistics of the functional score

Time Point	Group A (Mean ± SD)	Group A Median (Min–Max)	Group B (Mean ± SD)	Group B Median (Min–Max)	t-value	P-Value
At Baseline	61.55 ± 9.83	60.31 (38–77)	59.48 ± 9.56	57.76 (42–77)	0.95	0.345
At 5th Day	65.28 ± 9.53	64.65 (43–83)	63.43 ± 9.46	61.54 (42–83)	2.01	0.048
At 2nd Week	66.78 ± 9.61	66.28 (44–86)	65.02 ± 9.41	63.25 (44–85)	2.08	0.041
At 6th Week	72.65 ± 10.02	72.59 (52–95)	71.22 ± 10.03	69.92 (48–94)	2.11	0.037
At 12th Week	76.38 ± 10.37	76.90 (55–101)	75.18 ± 10.40	74.28 (50–101)	2.22	0.029

Association analysis demonstrated a meaningful relationship between the use of cryotherapy and improvements in multiple clinical outcomes. Pain levels, as measured by VAS, showed a stronger association with cryotherapy during the early and mid-recovery phases, while differences became less pronounced by the final follow-up. Similarly, the range of motion was more favourable in the cryotherapy group at several time points, particularly during the early and late stages of recovery. Both knee scores and functional scores were consistently associated with cryotherapy across all follow-up intervals beyond the baseline, reinforcing the therapy’s positive influence on postoperative performance. These associations support the conclusion that cryotherapy may contribute significantly to improved pain management, joint mobility, and functional outcomes in patients recovering from total knee arthroplasty.

## Discussion

Cryotherapy is widely gaining acceptance as a modality for orthopaedic postoperative practice, especially after interventions such as TKA, where inflammation, pain, and loss of movement substantially hamper recovery [23]. Cryotherapy, which lowers tissue temperature, reduces enzymatic activity, causes vasoconstriction, and impairs the transmission of nociceptive signals, may be theoretically well-suited to enhance early postoperative results. Although its benefit, particularly in minimally invasive surgery, has been debated in some study series [21], data from the present study indicate the importance of cryotherapy for TKA rehabilitation.

This study showed that the cryotherapy group had better pain management in the immediate postoperative period and faster knee function recovery compared to those receiving only standard care. The considerable decrease in VAS scores is also similar to the report of Morsi and Naidu et al., in which pain was significantly decreased in patients who used continuous flow cooling devices following bilateral TKA [24,25]. Similarly, Kuyucu et al. reported reduced analgesic requirements and pain scores in cryotherapy-treated patients, further supporting its role in multimodal analgesia protocols [26]. Good pain relief is fundamental to successful rehabilitation and permits early mobilisation, lowers the risk of postoperative complications, such as thromboembolism, and increases patient satisfaction.

Although pharmacological treatments are available for pain, they can have serious side effects. Opiates are the most frequently practised treatment but can result in sedation, respiratory depression, nausea, constipation, and addiction [27,28]. Nonsteroidal anti-inflammatory drugs (NSAIDs), which are also widely used in postoperative pain management, can lead to gastric irritation, peptic ulcers and bleeding, especially in elderly individuals or with the presence of comorbidities [29]. In this setting, cryotherapy seems to be an interesting non-pharmacologic add-on that could reduce the need for systemic medication and the associated side effects.

Improvement in ROM also seemed greater in the cryotherapy group during all follow-up periods. This aligns with Morsi et al.'s results, which also favoured the effect of cryotherapy on joint mobilisation after TKA [24]. Although Lessard et al. found no significant differences in ROM after arthroscopic knee surgery [21], the invasiveness and inflammatory load of TKA are much heavier and may justify the greater advantage seen in our sample. Although there was a statistically significant 3.35° difference in ROM between groups at 12 weeks, its clinical importance may be minimal and should be considered with the overall rehabilitation goals and patient function. These findings indicate that cryotherapy may be more effective in surgeries with extensive tissue trauma and inflammation.

In addition, significantly higher KSS and functional status were found in the cryotherapy group. These marginal benefits represent greater recovery in knee stability, alignment, and patient-reported function. Kuyucu et al. also observed a substantial improvement in knee scores in the treatment group with the application of cryotherapy, consistent with our findings. Although there were few differences in scores between the two groups, the general trend across all time points indicated that cryotherapy benefited rehabilitation, particularly in the early postoperative weeks.

Notably, our use of cryotherapy as part of the recovery protocol was not associated with side effects; however, specific subpopulations (e.g. patients with Raynaud’s phenomenon or cryoglobulinaemia) may require separate consideration because of possible risks of cold-induced tissue injury [30]. These safety concerns highlight the importance of proper patient selection before the use of cryotherapy.

Recent evidence supports the beneficial role of cryotherapy in TKA recovery such as Lee et al. It concluded that combining cryotherapy with early exercise significantly improved pain, swelling, ROM, and gait in post-TKA patients [31]. Chareancholvanich et al. compared three different techniques of cryotherapy and found that certain methods provided better pain control and patient satisfaction during the early recovery period [32]. Another study by Yang et al. evaluated the mechanisms, duration, and frequency of cryotherapy and added to the evidence that cryotherapy was effective in decreasing postoperative inflammatory response and swelling [33].

Additionally, Liu et al. did a meta-analysis of RCTs on the topic of continuous versus conventional cryotherapy and found no difference between the two approaches. Therefore, we would suggest that conventional options still are cost-effective and practical [34]. The review by Aggarwal et al. in Cochrane indicated slight reductions in blood loss, pain, and ROM by cryotherapy, while the authors highlighted the generally low quality of evidence and recommended additional high-quality studies [35].

Despite the supportive findings, there are several limitations that must be considered. This was an observational study and used a consecutive non-probability sampling method; therefore, there is a risk of selection bias. There was no randomisation or blinding, which could lead to performance and placebo effects. Baseline imbalances in age, weight, BMI, and comorbidity distribution (notably, higher diabetes prevalence in Group B and more chronic kidney disease in Group A) were observed. Despite post-stratification analyses, there are chances of residual confounding, which may affect functional outcomes. Moreover, in this study, the duration of the cryotherapy session was set at 20 minutes daily, which was practical yet different from durations used in previous research. Thus, it could impact comparative efficacy. The cryotherapy device used was a third-generation system, and the results may not be generalised to older and less sophisticated cooling technologies. The study also did not adjust for multiple comparisons across time points, raising the possibility of a Type I error. The single-centre setting also limits the generalisation of the results and cost-effectiveness in diverse healthcare environments. Notably, while pain resolution at 12 weeks was complete in the cryotherapy group, the difference in VAS was not statistically significant (p = 0.067), and this should be interpreted cautiously rather than as definitive evidence of superiority.

These limitations highlight the need for larger randomised controlled trials with standardised cryotherapy protocols and broader population samples to validate the clinical utility of cryotherapy post-TKA.

## Conclusions

This study suggests that cryotherapy may serve as a useful add-on in the postoperative management of total knee arthroplasty (TKA). Patients who were given cryotherapy had moderate but consistent improvements in pain, range of motion, and functional recovery compared to those who did not receive cryotherapy. These improvements were noted from the fifth postoperative day and continued over a 12-week follow-up period. At various time points, the observed differences were statistically significant, but effect sizes were modest and not uniformly sustained. Cryotherapy may be included in post-operative protocols, especially when early mobility and good pain control are priorities, because of its noninvasive nature and safe and easy usage.

## References

[1] Adie S, Naylor JM, Harris IA (2010). Cryotherapy after total knee arthroplasty a systematic review and meta-analysis of randomized controlled trials. J Arthroplasty.

[2] Gerbershagen HJ, Aduckathil S, van Wijck AJ, Peelen LM, Kalkman CJ, Meissner W (2013). Pain intensity on the first day after surgery. A prospective cohort study comparing 179 surgical procedures. Anesthesiology.

[3] Grosu I, Lavand'homme P, Thienpont E (2014). Pain after knee arthroplasty: an unresolved issue. Knee Surg Sports Traumatol Arthrosc.

[4] Holm B, Husted H, Kehlet H, Bandholm T (2012). Effect of knee joint icing on knee extension strength and knee pain early after total knee arthroplasty: a randomized cross-over study. Clin Rehabil.

[5] Sehat KR, Evans RL, Newman JH (2004). Hidden blood loss following hip and knee arthroplasty. Correct management of blood loss should take hidden loss into account. J Bone Joint Surg Br.

[6] Drez D (1990). Therapeutic modalities for sports injuries. Chicago: Year Book Medical Publishers Inc.

[7] Jutte LS, Merrick MA, Ingersoll CD, Edwards JE (2001). The relationship between intramuscular temperature, skin temperature, and adipose thickness during cryotherapy and rewarming. Arch Phys Med Rehabil.

[8] Levy AS, Marmar E (1993). The role of cold compression dressings in the postoperative treatment of total knee arthroplasty. Clin Orthop.

[9] Abramson DI, Chu LS, Tuck S Jr, Lee SW, Richardson G, Levin M (1966). Effect of tissue temperatures and blood flow on motor nerve conduction velocity. JAMA.

[10] Meeusen R, van der Veen P, Harley S (2001). Cold, and compression in the treatment of athletic injuries. Am J Med Sports.

[11] Nielsen HV (1983). External pressure--blood flow relations during limb compression in man. Acta Physiol Scand.

[12] Brodell JD, Axon DL, Evarts CM (1986). The Robert Jones bandage. J Bone Joint Surg Br.

[13] Saito N, Horiuchi H, Kobayashi S, Nawata M, Takaoka K (2004). Continuous local cooling for pain relief following total hip arthroplasty. J Arthroplasty.

[14] Barry S, Wallace L, Lamb S (2003). Cryotherapy after total knee replacement: a survey of current practice. Physiother Res Int.

[15] Cohn BT, Draeger RI, Jackson DW (1989). The effects of cold therapy in the postoperative management of pain in patients undergoing anterior cruciate ligament reconstruction. Am J Sports Med.

[16] Daniel DM, Stone ML, Arendt DL (1994). The effect of cold therapy on pain, swelling, and range of motion after anterior cruciate ligament reconstructive surgery. Arthroscopy.

[17] Melzack R (1975). The McGill pain questionnaire: major properties and scoring methods. Pain.

[18] Hecht PJ, Bachmann S, Booth RE, Rothman RH (1983). Effects of thermal therapy on rehabilitation after total knee arthroplasty. Clin Orthop.

[19] Walker RH, Morris BA, Angulo DL, Schneider J, Colwell CW Jr (1991). Postoperative use of continuous passive motion, transcutaneous electrical nerve stimulation, and continuous cooling pad following total knee arthroplasty. J Arthroplasty.

[20] Williams J, Harvey J, Tannenbaum H (1986). Use of superficial heat versus ice for the rheumatoid arthritic shoulder: a pilot study. Physiother Can.

[21] Belitsky RB, Odam SJ, Hubley-Kozey C (1987). Evaluation of the effectiveness of wet ice, dry ice, and cryogenic packs in reducing skin temperature. Phys Ther.

[22] Oosterveld FG, Rasker JJ, Jacobs JW, Overmars HJ (1992). The effect of local heat and cold therapy on the intraarticular and skin surface temperature of the knee. Arthritis Rheum.

[23] Schaser KD, Stover JF, Melcher I (2006). Local cooling restores microcirculatory hemodynamics after closed soft-tissue trauma in rats. J Trauma.

[24] Morsi E (2002). Continuous-flow cold therapy after total knee arthroplasty. J Arthroplasty.

[25] Naidu S, Baskerville PA, Goss DE, Roberts VC (1994). Raynaud’s phenomenon and cold stress testing: a new approach. Eur J Vasc Surg.

[26] Kuyucu E, Bülbül M, Kara A, Koçyiğit F, Erdil M (2015). Is cold therapy really efficient after knee arthroplasty?. Ann Med Surg (Lond).

[27] Goldhaber SZ, Bounameaux H (2012). Pulmonary embolism, and deep vein thrombosis. Lancet.

[28] Patel VP, Walsh M, Sehgal B, Preston C, DeWal H, Di Cesare PE (2007). Factors associated with prolonged wound drainage after primary total hip and knee arthroplasty. J Bone Joint Surg Am.

[29] Lotke PA, Faralli VJ, Orenstein EM, Ecker ML (1991). Blood loss after total knee replacement: effects of tourniquet release and continuous passive motion. J Bone Joint Surg Am.

[30] Herrera E, Sandoval MC, Camargo DM, Salvini TF (2010). Motor and sensory nerve conduction are affected differently by ice pack, ice massage, and cold water immersion. Phys Ther.

[31] Lee B, Yoon D, Yim J (2024). Effects of an early exercise program with cryotherapy on range of motion, pain, swelling, and gait in patients with total knee arthroplasty: a randomized controlled trial. J Clin Med.

[32] Chareancholvanich K, Keesukpunt W, Pornrattanamaneewong C, Narkbunnam R, Jarusriwanna A (2025). Comparison of three cryotherapy techniques for early post-TKA pain control in terms of efficacy and patient satisfaction: a randomized controlled trial. Arthroplasty.

[33] Yang L, Zhan YF, Zhai ZJ, Ruan H, Li HW (2025). Mechanisms and parameters of cryotherapy intervention for early postoperative swelling following total knee arthroplasty: a scoping review. J Exp Orthop.

[34] Liu MM, Tian M, Luo C, Wang S, Shao L (2022). Continuous cryotherapy vs. traditional cryotherapy after total knee arthroplasty: a systematic review and meta-analysis of randomized controlled trials. Front Surg.

[35] Aggarwal A, Adie S, Harris IA, Naylor J (2023). Cryotherapy following total knee replacement. Cochrane Database Syst Rev.

